# Maternal obesity support services: a qualitative study of the perspectives of women and midwives

**DOI:** 10.1186/1471-2393-11-69

**Published:** 2011-10-08

**Authors:** Penny J Furness, Kerry McSeveny, Madelynne A Arden, Carolyn Garland, Andy M Dearden, Hora Soltani

**Affiliations:** 1 Faculty of Health and Wellbeing, Sheffield Hallam University, Collegiate Crescent, Sheffield, S10 2BP, UK; 2 Communication and Computing Research Centre, Sheffield Hallam University, Arundel Street, Sheffield, S1 2NU, UK; 3 Department of Psychology, Sociology and Politics, Sheffield Hallam University, Collegiate Crescent, Sheffield, S10 2BP, UK; 4 Doncaster and Bassetlaw Hospitals NHS Foundation Trust, Armthorpe Road, Doncaster, DN2 5LT, UK; 5 Health & Social Care Research Centre, Sheffield Hallam University, Collegiate Crescent, Sheffield, S10 2BP, UK

## Abstract

**Background:**

Twenty percent of pregnant women in the UK are obese (BMI ≥ 30 kg/m^2^), reflecting the growing public health challenge of obesity in the 21st century. Obesity increases the risk of adverse outcomes during pregnancy and birth and has significant cost implications for maternity services. Gestational weight management strategies are a high priority; however the evidence for effective, feasible and acceptable weight control interventions is limited and inconclusive. This qualitative study explored the experiences and perceptions of pregnant women and midwives regarding existing support for weight management in pregnancy and their ideas for service development.

**Methods:**

A purposive sample of 6 women and 7 midwives from Doncaster, UK, participated in two separate focus groups. Transcripts were analysed thematically.

**Results:**

Two overarching themes were identified, 'Explanations for obesity and weight management' and 'Best care for pregnant women'. 'Explanations' included a lack of knowledge about weight, diet and exercise during pregnancy; self-talk messages which excused overeating; difficulties maintaining motivation for a healthy lifestyle; the importance of social support; stigmatisation; and sensitivity surrounding communication about obesity between midwives and their clients. 'Best care' suggested that weight management required care which was consistent and continuous, supportive and non-judgemental, and which created opportunities for interaction and mutual support between obese pregnant women.

**Conclusions:**

Women need unambiguous advice regarding healthy lifestyles, diet and exercise in pregnancy to address a lack of knowledge and a tendency towards unhelpful self-talk messages. Midwives expressed difficulties in communicating with their clients about their weight, given awareness that obesity is a sensitive and potentially stigmatising issue. This indicates more could be done to educate and support them in their work with obese pregnant women. Motivation and social support were strong explanatory themes for obesity and weight management, suggesting that interventions should focus on motivational strategies and social support facilitation.

## Background

Obesity (body mass index (BMI) ≥ 30 kg/m^2^) is identified as a major public health challenge of the 21st century across the globe [1]. In the UK, 23% of the population are obese and it is predicted that more than half of the adult population will be obese by 2050 [2]. Obesity is also a growing problem for women of childbearing age [3] and about one fifth of pregnant women in the UK are obese [4].

According to the Confidential Enquiry into Maternal and Child Health [5], obesity is associated with over half of the total maternal deaths from direct and indirect causes. Pre-pregnancy obesity and excessive weight gain during pregnancy are associated with adverse outcomes during pregnancy and birth [6-9], and with obesity in the offspring [10]. Excessive weight gain during pregnancy can increase the likelihood of further development of obesity, perpetuating the cycle of risk [11]. Postpartum body composition may be affected by gestation: increased fat retention has been observed in obese compared to normal weight mothers [12]. Maternal obesity also has psychosocial implications due to stigmatisation [13,14]. Obesity in pregnancy therefore has considerable implications for health service provision [15]: the cost of obese pregnancy care was estimated to be at least five times greater than that of normal weight mothers [6].

Pregnancy, however, can be seen as a naturally occurring opportunity to alter embedded attitudes and habits and adopt new activities [16-18] and, therefore, to address obesity. Lee & Koren [19] recommend pre-conception counselling to educate women planning pregnancy about the risks of maternal obesity and to encourage healthy lifestyles. Effective gestational weight management strategies are of high priority since they can reduce the likelihood of all the above complications and reduce the risk of further development of obesity in mothers and their offspring. Although behavioural and pharmacological interventions for obesity in the general population are well researched, there is relatively little research into efficacy of weight control interventions in pregnancy [20]. Published studies describe a range of supportive, educational and/or behavioural interventions to manage weight and encourage healthy diet and activity; however results are inconclusive (e.g. [21-23] comp. [24,25]). Systematic reviews [26,27] conclude that insufficient evidence exists about the efficacy of dietary and physical activity interventions in pregnancy. Oteng-Ntim et al. [28], who explored service providers' views regarding maternal obesity interventions, recommended that both service providers' and service users' perspectives should be sought when developing new services to ensure they are not only theoretically effective but also acceptable to those who will utilise them. Given the conflicting findings thus far, it is important to continue exploring and evaluating approaches to tackling maternal obesity and consider their acceptability to women and feasibility and cost-effectiveness in practice [28].

## Methods

### Aims

This study aimed to explore women's experiences of managing weight in pregnancy and the perceptions of women, midwives and obstetricians of services to support obese pregnant women in managing their weight. It also aimed to explore their perspectives upon the use of mobile technology in supporting obese pregnant women; however these results will be reported elsewhere.

### Design

This exploratory, qualitative study used focus group methodology to gather data from obese pregnant women (BMI ≥ 30 kg/m^2^) and practitioners delivering maternity services in Doncaster, UK, in 2011. In the light of existing evidence suggesting a strong association between obesity and pregnancy and birth complications, although many of the issues addressed may also be relevant to overweight women (BMI 25-30 kg/m^2^), the focus of this study is women with a BMI ≥ 30 kg/m^2^.

### Participants & Recruitment

Following University (Sheffield Hallam University Research Ethics Committee), NHS (South Yorkshire Research Ethics Committee) and local (Doncaster and Bassetlaw Hospitals Research and Development Department) ethical approvals, maternity service users (BMI ≥ 30 kg/m^2^) in Doncaster, and obstetricians and midwives from both community and hospital settings were approached to participate, using a purposive sampling strategy.

Doncaster is situated the North East of the United Kingdom. Ninety five percent describe their ethnicity as White. Around 38% of the female population of Doncaster is of child-bearing age (15-44), (compared to 40% in the UK as a whole) and this figure is predicted to rise by close to 10% by 2020. The general fertility rate (GFR) rose from 63.9 live births per 1000 women in 2006 to 68.1 in 2008, which is considerably higher than the UK average (63.9 in 2008); however, it is estimated that the number of births will fall by 5.4% by 2031 [29]. Doncaster has a population with high levels of socio-economic deprivation, which has been clearly linked to maternal obesity [15]. Life expectancy, infant deaths, deaths from smoking and people diagnosed with diabetes, are all worse than the England average and over one third of the local population fall into the 'most deprived quintile' of the UK [30]. In 2009, 24% of adults in Doncaster were obese and nearly 20% of women were obese at the beginning of their pregnancy. Tackling obesity is a top priority in the Doncaster region [30]. Doncaster maternity services run an innovative service for obese (BMI ≥ 30) pregnant women (known to users as 'Monday clinic'). Women referred to this service at their booking appointment enjoy additional midwife support and dietitian input, exercise opportunities, support groups and counselling, and can continue to access these services throughout pregnancy. The aims of the clinic are to encourage and support women to make lifestyle and behavioural changes in the antenatal period which are sustainable after they give birth [31,32].

Local midwives provided eligible women with a letter, information sheet and reply slips to register interest. Midwives were informed about the study via email. Responders were telephoned to make arrangements for participation, and answer any questions they had about the study.

A total of 6 women and 7 midwives were recruited to the study. No obstetricians responded to invitations to participate (despite offers of individual interviews at convenient times). Due to the qualitative nature of the study, specific demographic data were not collected; however it was evident from observation and through discussion that the women were all white, aged 18 to 40, and had experienced between 1 and 4 pregnancies. Midwives were all white, female and ranged from a few years' to decades of midwifery experience, which included antenatal, intrapartum and postpartum care. Within the sample, 1 midwife worked in the 'Monday clinic' and 2 women had been referred to and used this service.

### Data Collection

Semi-structured focus groups were used to collect data, whose aim was to facilitate interaction between participants in a permissive, comfortable environment [33]. In comparison with individual interviews, focus groups allow participants to share their ideas and experiences with one another as well as facilitators, which can broaden discussion. Focus groups enable individual perspectives to be aired before and discussed with others who have similar experiences, and can have a synergistic effect. They also reduce the 'voice' of the interviewer and emphasize the opinions and concerns of the group. Women and midwives participated in separate focus groups to eliminate the possible effect of hierarchy on discussion. Participants in each group had much in common including potentially sensitive issues such as the experience of being obese, encountering negative social responses, or caring for obese clients. Holding separate groups reduced the potential for confrontation and distress which can arise when people with different experiences and conflicting perspectives are brought together. Each lasted around 1 hour and numbers (n = 6 and 7) were considered optimal in generating a range of ideas and maximising involvement in discussion of this sensitive subject. Both groups were held in a local Children's Centre, a familiar environment chosen to provide a welcoming, neutral context for discussions.

Women were asked about their experiences of weight management in pregnancy, their opinions about existing maternity services and their ideas about effective support for obese pregnant women. Midwives were asked about their experiences of caring for women who had difficulties managing their weight, the types of support currently offered for obese women and to comment upon women's ideas for effective support. Facilitators (PF & KM) guided the conversation but were otherwise minimally involved to ensure participants' views were predominant [34]. Focus group discussions were audio-recorded with participants' consent.

### Data Analysis

Focus group data were transcribed and anonymised: all participants were allocated a pseudonym. Transcriptions were loaded into NVivo 8, a software package which assists in qualitative data analysis by facilitating storage, organisation and retrieval processes. Data analysis was an inductive process of thematic analysis [35] which comprised careful reading and coding of all data, generation of categories of related data and development of overarching themes. For the purpose of inter-rater reliability, initial analysis was conducted independently by two researchers (PF & HS), who agreed emerging themes. Analysis was verified by a third researcher (KM) and then discussed with and approved by the team.

## Results

Two overarching themes were identified in the data: (1) Explanations for obesity and weight management and (2) Best care for overweight women. These themes and subthemes are explained with illustrative quotations from participants.

### Overarching theme 1: Explanations for obesity and weight management

Participants discussed at length factors they believed contributed to obesity and weight management in pregnant women. The four key subthemes were 'Information, knowledge and skills', 'Psychological and lifestyle factors', 'Stigma' and 'Communication'.

#### Information, knowledge and skills

These women understood that eating and activity were related to weight and health; however they lacked confidence about intake requirements, food safety and appropriate levels and types of exercise in pregnancy. This confusion was exacerbated by what they perceived as ever-changing media messages and a lack of nutritional advice:

*The first time I was pregnant was 4 years ago now ... I put on about 4 stone then. I just piled it on because I didn't have the support, and I didn't know what to eat, how much I should be eating or anything like that; if I could exercise still when I was pregnant: I didn't have anything *(Sally).

Midwives also felt some of their clients lacked the knowledge and skills to maintain a healthy lifestyle. In particular, they perceived that obese pregnant women '*don't seem to realise the implications of their high BMI*' (Becky, MW), did not understand healthy eating and lacked cooking skills. Although the women and midwives in this study identified gaps in women's knowledge and skills, their perspectives differed somewhat: the women here felt the gap was in the information they received, whereas midwives felt that general awareness and skills were lacking. These varying perspectives may help explain why the women in this study felt they weren't getting the information they required. Alternatively the difference may reflect a discrepancy between the women who participated and the larger group of women cared for by the seven participating midwives. When asked how typical their views and experiences were, these women felt they were representative of their peers; however midwives believed that the participants represented the keener, proactive end of the spectrum of women under their care.

#### Psychological and lifestyle factors

This was a dominant explanation for obesity in pregnancy for both women and midwives. Key issues raised were self-talk, motivation and social support.

##### Self-talk

Women identified internal dialogues regarding eating in pregnancy, sometimes influenced by social messages. Self-talk messages included using pregnancy as a reason to overeat, overeating once morning sickness had passed 'to make up for' previous eating problems, and assuming weight gain in pregnancy would be quickly lost afterwards, especially when breastfeeding. Postpartum, some women here said they continued to treat pregnancy as an 'excuse' for months or years for obesity:

*I think there is only so long that you can get away with, 'yeah, I'm this fat, I've just had a baby' - she's 6 months old now. I think I can't really get away with saying that for much longer, do you know what I mean? Yeah 'I've just had a baby' and soon it will be that she's 4, and it just won't have gone *(Lucy).

It seemed these women were not fully convinced by their self-talk messages; underlying the 'excuses' lay an awareness that they were unrealistic and would at some point have to be put aside. This suggests that some input from midwives, health visitors, GPs or obstetricians could help obese women tackle their unhelpful self-talk and provide support to make the necessary changes at an earlier stage.

##### Motivation

A linked issue was motivation, raised by women and strongly confirmed by midwives:

*I think sometimes it is a motivation issue. Although they would like to lose the weight, they don't want to put the work in to actually do that. They think it's something that's not achievable, or they are just not motivated for it *(Sarah, MW).

In keeping with this view, women participants reported struggling to find the motivation to exercise, alter eating habits, and maintain positive efforts over long periods of time, and '*getting carried away*' (Jenny) with eating. They shared sympathetic laughter with those who related stories of trying unsuccessfully to resist temptation:

*I think you should probably be motivated enough yourself, knowing that you've got to lose weight before your holiday or whatever, but it's just, for me, I've got no willpower. I will just end up with the bar of chocolate in front of the TV, rather than going out (general laughter) *(Lucy).

Although aware of individual variation, some midwives believed healthy weight was simply not a priority for many of their obese pregnant clients:

*I think many of them are aware of diet and the implications, but I don't actually think they care, in all honesty, a lot of them. I work in an area that has a lowish socioeconomic group, lots of smokers, lots of teenage pregnancies, high BMIs, all the things that go with that, and I don't think it's a priority in their lives, to be truthful *(Sarah, MW).

There was general agreement that the focus group women were likely to be '*quite motivated and quite positive*' (thus not representative of those referred to by Sarah, above). Some perceived that motivation was inversely related to body weight, with moderately obese clients easier to motivate than those with higher BMIs.

*I've found that the women who seem to be most concerned are those with a BMI between 30 and 35. 'I know I'm a bit podgy, I know I'm a bit big, and I don't want to get any bigger'. I wonder whether the larger women have just given up, and think 'I don't think you can help me because I can't help myself'. But I get a lot who are more receptive with a BMI of about 30, 32. They say, 'Oh yeah, I need help, I don't want to get any bigger' *(Jackie, MW).

Pregnancy and motherhood were considered as theoretically good opportunities to support women with BMI ≥ 30 kg/m^2 ^in behaviour change due to potential triggers such as struggling to carry a baby upstairs, keep up with their children, and fears about school bullying. Nonetheless midwives reported feeling at a loss regarding how to motivate obese women and described admitting defeat in certain cases:

*It's almost like they've given up on themselves, and you can hear yourself, that you've given up on them. But then, what more can we do, we can't, it's absolutely true, but isn't it sad, we've given up on them too *(Jackie, MW).

##### Social support

The women here reported feeling lonely and isolated at times. Unhealthy eating and inactivity seemed more likely when they were alone, but others' support helped motivate them to eat well and become more active:

*I used to be so tired after work and think I can't be bothered to go [to Aquanatal] ... And that sort of motivated me to get up and go, knowing that everyone was gonna be there, and everyone else was gonna be the same as me *(Lucy).

#### Stigma

The stigma of obesity had different meanings for women and midwives. Midwives, for example, discussed how attitudes towards weight had changed over time, the greater acceptance of obesity, and the relative ease today of finding fashionable clothing in larger sizes. They felt these social changes meant larger women were still able make positive social comparisons and were less motivated to heed midwives' advice to alter health behaviours and manage weight.

*I think that our idea of what's big has changed, and so it's more acceptable to be bigger because there are more people... They can buy clothes easier. It's not so difficult for them to buy clothes and be accepted as what it was. So I think that when they come to clinic at first ... They know we're going to mention it, and then they just quickly want to get it over with, they nod their head when we talk about being referred [to Monday clinic], but 'let's move on and talk about the baby *(Jackie, MW).

Women in this study had a different view. Although aware of changing attitudes, they felt stigmatised due to their weight and vulnerable to negative attitudes and judgements nonetheless:

*I think the stigma is that if you're over a certain BMI that you don't exercise isn't it? (General agreement) That's what people think. I mean if you're slim and you've got a low BMI then they automatically think that you exercise, if you're not then they think you don't *(Kate).

Women here reported embarrassment about their weight during and after pregnancy and feeling conspicuous in social situations:

*I think you just feel like, because you are pregnant, you're fat anyway, and being big before, you feel like everybody is looking at you. You don't want to go anywhere; I got to a stage where I didn't want to go out of the house *(Rachel).

Some women attending a general weight loss class postpartum had felt obliged to explain to others that they had been pregnant in order to justify their present size and weight. They preferred weight-management activities alongside other mothers because '*everyone's in the same boat' *(Lucy), believing other people did not understand how they felt.

This difference in attitudes suggests that midwives may inadvertently make assumptions about women's response to their size and underestimate the pressures upon them. This could limit their ability to understand and respond to the psychosocial consequences of obesity for women, such as those discussed above.

#### Communication

The stigma of obesity seemed to create problems for communication between midwives and pregnant women. As one midwife commented, '*It's quite acceptable now to talk to women about smoking, but it's still not quite acceptable to say to a woman, 'your weight may kill your baby'*' (Anna, MW). Jackie (MW) observed '*you're not allowed to use the F word, are you, the fat word?*' As a result of sensitivity surrounding language and anxiety about creating offence, it was perceived that women's obesity may be '*skirted around*', not acknowledged properly during midwife consultations, and that messages were given in a vague, indirect manner to '*protect*' both parties (Anna, MW):

*I have a disk that I work out people's BMIs on, and it says 'obese' there, and I can't say it; I say to them 'well this is where your BMI is, look.' And I've said it, but she can't say 'she called me obese', but I say 'look, look, you're there look, that's what it says you are'. So I'm anxious, but I'm also protecting myself, y'know, and we don't use the language that we should be using sometimes, do we? *(Jackie, MW).

Despite midwives' reluctance to communicate openly, the women in this study were aware that their obesity was a cause of concern and resulted in extra tests and referrals. Midwives' concerns appeared justified by the attitude of at least one of the women in the focus group, however, who did not want her pregnancy care dominated by her weight:

*I felt again like I was being penalised because I was fat. I used to say, 'oh, I've got to do the fat girls' test again, have I?' All the time, I felt like they were picking on you because you were fat ... But that's obviously because I knew I was fat, and I was just 'I don't need somebody to tell me that I'm fat, thank you very much!' *(Alice).

According to these findings, messages about obesity and weight management in pregnancy at present may be blurred by unspoken anxieties and resentments. These comments suggest more work needs to be done to overcome social barriers which affect midwives' confidence in raising the issue of weight and discussing its implications with women in their care.

### Overarching theme 2: Best care for overweight women

The women participants had varying experiences of midwife and obstetric care. The discussed what they perceived as more or less helpful about that care and its implications for weight management. Here subthemes comprised: 'Consistency and Continuity', 'Support not judgement', and 'Opportunities for interaction'.

#### Consistency and continuity

Continuity of care meant seeing the same midwives repeatedly, so that a rapport could be developed, as well as hearing clear messages about pregnancy and weight. Most of these women had seen many different midwives in pregnancy so hadn't enjoyed the type of relationship in which discussions about weight management could develop:

*I used to see a different midwife every week, so I was gaining a lot of weight and not really talking to anybody about it. I think if I'd have seen the same midwife all the way through and talked to someone about healthy eating and stuff like that, it would have been easier, because I gained about 4 stones in total *(Jenny).

Women also noted that the messages they received about weight gain in pregnancy were inconsistent. Several reported trying to get advice about their weight from the midwives they saw; however they felt midwives' responses were vague and did not reinforce the importance of weight or women's efforts to manage it:

*I had mentioned it a few times and they were like 'oh, well, you're pregnant; you are going to put weight on' *(Jenny).

*I used to ask to get weighed because I was really worried about putting weight on, and the midwives told me, 'oh, we won't weigh you every time you come, you don't need to get weighed' *(Lucy).

*I was forever asking the midwives [about exercise activities] but I got swapped between a few midwives ... so I kind of found out there wasn't much information out there *(Rachel).

Clearly the women in this study felt they were receiving mixed messages and needed unambiguous advice in order to feel fully informed and supported in their weight management efforts. The midwives themselves believed they were giving clear, consistent information to women about being healthy, eating well and being active; however it appeared from speaking with the pregnant women that these messages were not getting across effectively.

#### Support not judgement

Some women reported unfortunate experiences of care in earlier pregnancies:

*There was one consultant that I used to go to, and he was a brilliant consultant but he had no bedside manner at all, and he was horrible. He used to have me in tears - every time I'd go and see him, he'd tell me I was putting on too much weight, and he would literally shout at me. I don't smoke, I've never drunk throughout; it was the only thing that I was doing wrong, and he used to have me in tears *(Sally).

Sally felt judged, stigmatised and unsupported by this obstetrician. In contrast, those women, including Sally on her most recent pregnancy, who attended the 'Monday clinic', were delighted with midwives' constructive, non-judgemental attitude, the provision of dietary advice and physical activity programs. Women were able to meet the same midwives each time, which promoted relationship-building and trust. This, in combination with the practical strategies and support helped motivate the women, and the resulting successes gave them a real sense of achievement:

*They were brilliant, and I put on just over a stone this time, which I lost within a day of having my son! So it was completely different, and I saw a dietitian, and I saw [instructor at local gym], and she set me out a program, so I could still go to the gym throughout it. It was just brilliant, and it was so different this time 'round, I can't explain how good it was! I'd get pregnant again just 'cause it was so good, whereas I was dreading it after the first time! It was brilliant *(Sally).

A positive feature of the 'Monday clinic', according to those who had attended, was its clear focus: '*I have noticed this time around that they are more about the weight, do you know what I mean? Everything is about the weight, compared to other pregnancies*' (Kate). This was positive, however, because it wasn't '*like I was going to be the worst mum ever because I was overweight*': instead '*they just want to help you, and they've been nice about it*' (Sally). Thus, although women were in no doubt as to why they were there, they appreciated the consistent care, clear advice and non-judgemental support.

#### Opportunity for interaction

In keeping with the importance of social support in motivating women and reducing the sense of isolation mentioned above, participants here believed they benefited most from interaction with midwives and other pregnant women. Some who had had their babies recently were now attending a postnatal exercise group or had been to Aquanatal during their pregnancies. Specifically designed sessions provided an opportunity for mutual support and healthier lifestyle in a non-judgemental, non-threatening environment: '*we all get to see each other every week, and you get to talk about how much you've lost or what you've done. That is nice, to be able to talk to other people in the same boat as you*' (Sally).

When asked to imagine ideal maternity care, these women wanted more opportunities to meet and support one another with healthy lifestyles, online and in person: '*Something with loads of other new mums, or going- to- be new mums, to bond for a day. To have a day of fun exercise-type activities, with like a bit of dieting*' (Rachel). When one suggested regular midwife-led walks for pregnant women, the others enthusiastically agreed. Some midwives were cautious about this idea, identifying barriers such as lack of midwife time and funding, and mobility or motivational variation in the service user group. Others, however, were keen, discussing similar previous initiatives which '*had worked really well ... because everyone was on board locally we got great numbers*' (Kathy). Jackie also noted that they had given midwives excellent opportunities for '*health promotion and advice*'.

## Discussion

This study explored the views of women and midwives about services and support for obese pregnant women in Doncaster, UK, a relatively deprived area with high levels of obesity in pregnancy. Results raised a number of issues, including a range of explanations for obesity and aspects relating to effectiveness of care for obese pregnant women.

Women reported a lack of confidence about what foods they should eat, how much and what types of exercise are safe, and how much weight gain is acceptable. The women felt they did not always receive clear guidance and that social messages, especially about eating, placed conflicting pressures upon them. These findings are mirrored in the UK and elsewhere. For example, Curzik et al. [36] comment that, although women are socialised to 'eat for two' there is a strong social pressure, reinforced by media images, to remain thin during pregnancy. A US survey [37] found that perceptions of acceptable weight gain in pregnancy vary greatly, with thinner women typically underestimating, and heavier women overestimating, recommended levels. Many women received no or inappropriate advice about weight gain, and half of overweight women were advised to gain more than recommended, despite established US guidelines [38]. Oteng-Ntim et al.'s UK service providers [28], like the women here, considered verbal advice offered to women on these topics was often inconsistent and unsupported by written information, a finding repeated elsewhere [39]. This is perhaps unsurprising, given the lack of UK guidance regarding appropriate weight gain in pregnancy [40]. There is also a widely held belief that breastfeeding protects against weight retention after pregnancy [39,41], which is not consistently supported by research evidence [42-44], possibly through early discontinuation of or overeating during breastfeeding [45]. This common misconception formed part of these women's self-talk which acted to limit their self-control of eating and activity during pregnancy. This suggests midwives need to provide clear advice to women early in pregnancy, backed up by written information and regular reinforcement, about healthy lifestyle in pregnancy, and that breastfeeding is unlikely to contribute to weight loss after pregnancy unless the woman is also active and eating healthily. The potential for UK midwives to offer specific advice about weight gain is currently limited by the absence of clear national guidelines [40].

Another finding from this study was that women's and midwives' perceptions about the psychosocial consequences of obesity differed. The women here were very aware of and often embarrassed by their weight. This contrasts with Olander et al.'s participants, who expressed little concern about weight gain [39]; however those women were not recruited on the basis of weight, hence obesity may not have been the pressing issue it was for the women in the present study. Smith et al. [46] note that body image is a concern for women both during and after pregnancy, especially postpartum with the social expectation that pregnancy weight will be lost, and women feel vulnerable to negative judgements [47]. Midwives believed that stigma surrounding obesity had reduced and, with it, the pressure to strive for a healthy weight. Alongside this, however, midwives' difficulties raising the issue of obesity with their clients, and awkwardness and anxiety around use of obesity-related language demonstrated that the stigma was still very much alive. Service providers elsewhere [28] raised very similar issues. Whereas these midwives erred on the side of caution, some women had clearly encountered practitioners whose critical and offensive approach had caused considerable distress. Findings here indicate that practitioners involved in pregnancy care may find it difficult to find ways to talk openly and honestly about obesity without causing offence to their clients. Concerns about the sensitivity of this issue are raised in other studies [48]. This suggests more could be done to raise awareness among student and practising midwives of the importance of obesity among women as both a physical and a psychological health issue, and to enhance their communication skills and confidence in discussing it effectively with service users. Those women who had attended the 'Monday clinic' appreciated and benefited from the clear, non-judgemental approach: specialist midwives could perhaps do more to disseminate their experience and expertise to their colleagues, and support them in this challenging aspect of their care. This small explorative study suggests that supportive, multidisciplinary innovations like Doncaster's 'Monday clinic' could be a valuable addition to maternity services elsewhere.

Factors considered by these women and midwives to influence eating and activity levels in pregnancy included motivation, and social support. A survey of 1535 pregnant US women found that barriers to exercise were most commonly intrapersonal, particularly motivation, procrastination, and a lack of time [49]. To date, intervention studies focusing upon dietary advice and exercise activities have shown inconsistent or limited effectiveness, especially with overweight women (e.g. [23,50]), which suggests that advice and provision of activities alone are not sufficient to address the problem in the long term. Oteng-Ntim et al. [28] identified client motivation and readiness to change as barriers to the effectiveness of service providers' efforts to promote healthy lifestyles for pregnant women. Motivation to act was a strong theme in the explanatory models of both midwives and women in these focus groups, which suggest more should be done to motivate obese pregnant women to make healthy lifestyle changes. This would clearly require additional investment in order to train and resource maternity service teams with the time and skills to deliver these services.

Women in this study felt at times isolated and expressed the need for more support from peers and professionals. Among interpersonal barriers identified by Evenson et al., lack of social support (informational, emotional and tangible) was most important [49]. Social support is considered one of the key influences upon and motivators for physical activity and healthy lifestyle changes, especially for women [51,52]. This indicates the importance of taking a holistic approach to midwifery care, considering the woman's social support network and influences and including family members in consultations where appropriate and consented. Given the importance, raised here and in other studies, of social support in motivating and supporting obese pregnant women in healthy lifestyle choices, interventions should consider how to harness this factor. Women here enjoyed meeting other pregnant women through existing services such as Aquanatal, but identified other possibilities such as organised walks, regular support days, and dedicated websites and chat rooms. This topic will be raised in another paper; however there is evidence from the general population that mobile technologies can be used very effectively to support healthy behaviour change and weight loss [53].

### Limitations

This was a small-scale, localised study with a qualitative methodology. Participants' perceptions and experiences may not reflect those of midwives and service users elsewhere, although similar findings have recently been made in another UK study [39]. Generalisability is rarely a priority within qualitative research; however, evidence from previous research suggests these are not isolated findings.

Results are limited by the participation of midwives alone from the health care team. Obstetricians did not respond to efforts to engage them, possibly due to workload and time constraints. It should also be acknowledged that a range of practitioners, including dietitians, are involved in the care of obese pregnant women. Failure to engage medical practitioners in research with a multi-professional focus is not unusual. A recent practice-based interprofessional learning project involving one of the authors had similar problems in some clinical areas [54,55]. In that case, it seemed that a lack of time and a tendency to prioritise projects with immediate relevance to their medical/surgical practice were the issues. It is not yet clear whether obstetricians we tried to include in this study had similar concerns.

Women interviewed here may not have been representative of other pregnant women in this locality; however midwives' perspectives were based on a more mixed client group and presenting the two alongside one another raises a number of issues for discussion. Women identified shortcomings and excellence in their care experiences and suggested ideas for improved service provision. More research is required to develop and trial these approaches to assess their feasibility, acceptability and effectiveness in practice. Furthermore, if successful in obese women, application of such services for women in other BMI groups (including overweight or normal weight) may also merit investigation. The decision was made to focus this study upon obese (rather than overweight women of BMI 25-30 kg/m^2^) because of the clear link in the literature between obesity and complications during pregnancy and birth. However it is arguable that issues raised by women and midwives in this study have relevance to women with lower BMIs and that future work should take a more inclusive approach.

### Implications

Midwives and health professionals may underestimate the considerable social stigma of obesity for the pregnant and postpartum woman. As well as encouraging healthy lifestyle choices, they should be aware of the psychosocial impact of obesity, be prepared to offer psychological support to avoid women feeling isolated, and take a constructive, non-judgemental approach to care. Midwives here reported struggling to talk to women openly about their weight, which suggests educators and experienced practitioners may need to consider how best to prepare and support staff caring for this client group.

Literature suggests more research is required to identify effective interventions for weight management among obese pregnant women. The midwives and women here independently identified that, among others, knowledge, motivation and social support are key factors in both causing and managing obesity in pregnant women. These perceptions are supported by findings elsewhere. This suggests that any intervention aimed at addressing maternal obesity should take account of these factors. Midwives and other health professionals caring for obese pregnant women should ensure messages about eating and exercise in pregnancy are consistent and unclouded by social misconceptions. Information alone may be insufficient to change long-standing behaviours and so interventions will need to address how to instigate and maintain motivation for health behaviour change in obese women. Health professionals and researchers should also consider how to harness natural social interaction opportunities and encourage social support for and between these women to help keep them engaged and motivated with healthy living plans.

## Conclusions

The findings suggest that obese women need unambiguous advice regarding healthy lifestyles, diet and exercise in pregnancy to address a lack of knowledge and a tendency towards unhelpful self-talk messages. Midwives expressed difficulties in communicating with their clients about their weight because of the sensitivity of the issue and concerns about causing distress. Given women's need for clear information and support from professionals, more could be done to educate, prepare and support midwives in their work with obese pregnant women. Motivation and social support were strong explanatory themes for obesity and weight management, suggesting that interventions to promote healthy lifestyles in pregnancy should focus on motivational strategies and social support facilitation.

## Declaration of Competing interests

The authors declare that they have no competing interests.

## Authors' contributions

PF collected and analysed data, drafted, revised and submitted the manuscript. KM collected and analysed data and helped revise the manuscript. MA participated in study design, data analysis and helped revise the manuscript; CG participated in study design, participant recruitment, data analysis and manuscript revision; AD participated in study design and coordination and contributed to data analysis and manuscript revision; HS was project lead, and was involved in all stages of study design and coordination, data analysis and manuscript development. All authors read and approved the final manuscript.

## Authors' information

PF (RN, BA Hons, MSc, PGCHCE, PhD) is a registered nurse, senior lecturer and research fellow in the Faculty of Health and Wellbeing. Her research interests include the psychology of health and illness, health promotion, and education (especially interprofessional) of healthcare students.

KM (BA Hons, MA, PhD) is a research assistant in the Communication and Computing Research Centre at Sheffield Hallam University. Her research interests include language and gender, the discursive construction of social issues, and the management of identity in talk about food and the body.

MA (BSc, PhD, C.Psychol) is a Principal Lecturer in Psychology at Sheffield Hallam University. She is a Health Psychologist and Chartered Psychologist with a particular interest in health behaviour change and women's health.

CG (RGN, RM, BMed Sci Hons) has been a practising midwife for fifteen years and a specialist in maternal obesity since 2008. She has jointly initiated an award winning midwifery-led clinic which supports obese pregnant women, and which has been recognised by the National Institute for Clinical Evidence on both their shared learning database and in their commissioning guide for Public Health Guidance 27.

AD (BSc, MSc, PGCE, PhD) is Professor of Interactive Systems Design at Sheffield Hallam University, UK. His research deals with ways of using interactive computer and communications systems to support social and economic development of communities. He is theme lead on User Centred Healthcare Design part of the NIHR funded Collaboration for Leadership in Applied Health Research and Care in South Yorkshire (CLAHRC-SY). He is co-chair of the International Federation for Information Processing's special interest group 13.1 on Interaction Design and International Development.

HS (PhD, MMedSci, BSc, PgDip, RM) is a Reader and the Lead for Service Delivery and Commissioning theme in the Health and Social Care Research Centre in Sheffield Hallam University, UK. She has been contributing to practice, education and research in midwifery for about 20 years. She is a member of Editorial Boards for maternity related journals, Research Standing Committee (RSC) for the International Confederation of Midwives (ICM) and Yorkshire &Humber Research for Patient Benefit Funding Committee.

## Pre-publication history

The pre-publication history for this paper can be accessed here:

http://www.biomedcentral.com/1471-2393/11/69/prepub
